# Personal receptor repertoires: olfaction as a model

**DOI:** 10.1186/1471-2164-13-414

**Published:** 2012-08-21

**Authors:** Tsviya Olender, Sebastian M Waszak, Maya Viavant, Miriam Khen, Edna Ben-Asher, Alejandro Reyes, Noam Nativ, Charles J Wysocki, Dongliang Ge, Doron Lancet

**Affiliations:** 1 Department of Molecular Genetics, Weizmann Institute of Science, Rehovot, 76100, Israel; 2 Institute of Bioengineering, School of Life Sciences, École Polytechnique Fédérale de Lausanne, Lausanne, 1015, Switzerland; 3 Genome Biology Unit. European Molecular Biology Laboratory, Meyerhofstrasse 1, 69117, Heidelberg, Germany; 4 Monell Chemical Senses Center, 3500 Market Street, Philadelphia, PA, 19104, USA; 5 Center for Human Genome Variation, Duke University School of Medicine, Durham, NC, United States of America

**Keywords:** Olfactory receptor, Genetic polymorphism, Haplotypes, Single nucleotide polymorphism, Copy number variation, Olfaction, Gene family

## Abstract

**Background:**

Information on nucleotide diversity along completely sequenced human genomes has increased tremendously over the last few years. This makes it possible to reassess the diversity status of distinct receptor proteins in different human individuals. To this end, we focused on the complete inventory of human olfactory receptor coding regions as a model for personal receptor repertoires.

**Results:**

By performing data-mining from public and private sources we scored genetic variations in 413 intact OR loci, for which one or more individuals had an intact open reading frame. Using 1000 Genomes Project haplotypes, we identified a total of 4069 full-length polypeptide variants encoded by these OR loci, average of ~10 per locus, constituting a lower limit for the effective human OR repertoire. Each individual is found to harbor as many as 600 OR allelic variants, ~50% higher than the locus count. Because OR neuronal expression is allelically excluded, this has direct effect on smell perception diversity of the species. We further identified 244 OR segregating pseudogenes (SPGs), loci showing both intact and pseudogene forms in the population, twenty-six of which are annotatively “resurrected” from a pseudogene status in the reference genome. Using a custom SNP microarray we validated 150 SPGs in a cohort of 468 individuals, with every individual genome averaging 36 disrupted sequence variations, 15 in homozygote form. Finally, we generated a multi-source compendium of 63 OR loci harboring deletion Copy Number Variations (CNVs). Our combined data suggest that 271 of the 413 intact OR loci (66%) are affected by nonfunctional SNPs/indels and/or CNVs.

**Conclusions:**

These results portray a case of unusually high genetic diversity, and suggest that individual humans have a highly personalized inventory of functional olfactory receptors, a conclusion that might apply to other receptor multigene families.

## Background

Olfaction, the sense of smell, is a versatile and sensitive mechanism for detecting and discriminating thousands of volatile odorants. Olfactory recognition is mediated by large repertoires of olfactory receptors (ORs), which activate a G-protein-mediated transduction cascade, located in the cilia of olfactory sensory neurons [1,2]. The human OR repertoire has 851 loci, encompassing 78 genomic clusters and 57 singleton loci, residing on all but two human chromosomes [3-6]. Each sensory cell expresses a single allele of a single OR locus, thus transmitting a molecularly defined signal to the brain [7-10]. A single OR gene may recognize more than a single odorant molecule [11-15]. A widely accepted working hypothesis is that allelic variants of OR genes may harbor different functional characteristics and hence, may generate different odorant sensitivity phenotypes in different members of the human population [16-18].

Human ORs encompass a high number of pseudogenes, whereby more than 50% of the loci annotated as nonfunctional due to frame-disrupting mutations [3,5,6,19]. Primates are less dependent than mouse and dog on olfactory cues, which appears to have resulted in a gradual gene loss process along this lineage [20-22]. Similar OR repertoire diminutions have been reported in other mammals [23]. In higher apes, the gene loss has remarkably accelerated in humans [24]. Such diminution of the functional OR repertoire in humans is an ongoing evolutionary process, as demonstrated by the past identification of OR genes that segregate between intact and pseudogene forms [25,26], and by more recent surveys showing an enrichment of loss-of-function OR alleles [27,28]. It was shown that every human individual is characterized by a different combination of such segregating pseudogenes (SPGs), constituting a pronounced genotypic diversity in the population, including ethnogeographic differences [26]. More recently, using a high-resolution microarray applied to 20 individuals [29], and a read-depth-based Copy Number Variation (CNV) genotyping algorithm [30], we showed a wide range of copy-number values across individuals, ranging from zero to nine copies. These results are in-line with other surveys which found a significant enrichment of ORs in CNV regions [31,32]. CNVs involving deletions (copy numbers of 0 or 1) were shown to affect 56 intact OR loci, 14% of the human OR gene repertoire [30].

Cell-surface receptors are often characterized by several haplotypic alleles in the population, sometimes with different functional properties. A prominent example is the group of the major histocompatibility proteins with varying specificities towards antigenic peptides [33,34]. Other examples include the taste receptor TAS38, underlying responsiveness to the bitter compound phenylthiocarbamide (PTC) [35,36], the melanocortin 1 receptor (MC1R), affecting human skin and hair pigmentation [37], and the green opsin OPN1MW, mediating red-green color vision discrimination [38]. Likewise, in the olfactory system, two protein haplotypes of the olfactory receptor OR7D4 were shown to manifest large difference in sensing the steroid odorant androstenone [39,40].

Some missense haplotypic alleles can be nonfunctional, due to a substitution of key amino acids governing protein folding or interaction with signal transduction components. A continuous spectrum of functionality among missense haplotypes may be quantified by algorithms such SIFT [41] or PolyPhen [42]. An analogous algorithm, Classifier for Olfactory Receptor Pseudogenes (CORP) [43], was previously used to identify 30 SNP variations for which one of the alleles is likely inactive [26], with a broader estimate of as many as 135 functionally inactive missense alleles in the reference genome [43].

Here, we performed scrutiny of publicly available data to create a comprehensive catalog of genetic variability in the human OR repertoire. This includes a compendium of all available missense haplotypes of OR proteins and a dramatically expanded list of OR segregating pseudogenes. Our work creates a framework for understanding the evolution and function of OR genes, and a necessary infrastructure for genotype-phenotype association studies for smell deficits. It further highlights the utility of the olfactory system as a model for personalized gene repertoires.

## Results

### Numerous allelic variants in intact ORs

**Figure 1:**
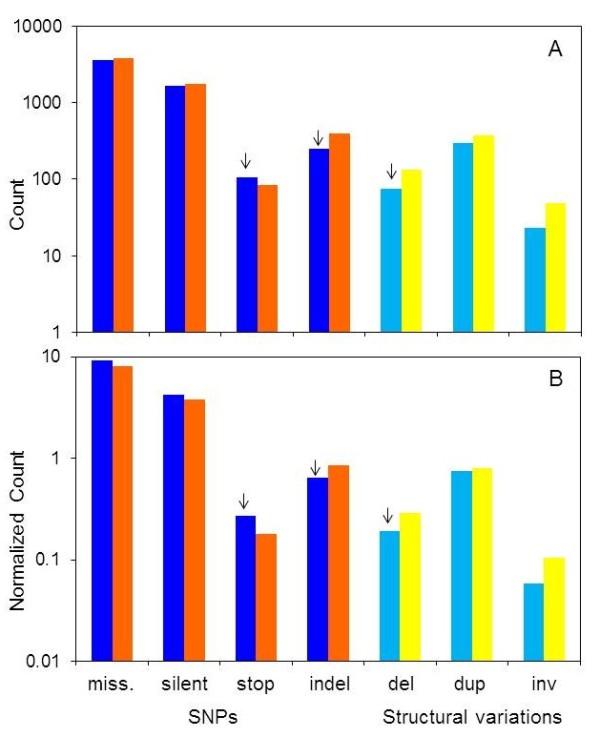
**A summary of the genomic variation counts in intact OR coding regions and in OR pseudogenes.A**, The absolute count. **B**, Count normalized per gene. Intact genes, blue and light blue; pseudogenes, orange and yellow. Nonfunctional variations are indicated by arrows. Abbreviations: miss, missense SNP; indel, small insertion/deletion up to 100 bp; del, CNV deletion; dup, CNV duplication; inv, CNV inversion; stop, stop gain/stop loss/ loss of the initiating methionine.

We performed *in-silico* data mining of genomic variations in OR genes and segregating pseudogenes, including single nucleotide polymorphisms, small indels (< 100 bp) and structural variations. These were obtained from 651 individuals of the 1000 Genomes Project, including three major ethnic groups, as well as from 11 additional resources (Additional file 1: Table S1). Our compendium contains 5,958 polymorphic events (variations) within coding regions of 413 functional gene loci, the latter selected as having an intact open reading frame in at least one of the individual human chromosomes analyzed (including 26 “resurrected” loci, see below). The breakdown of these variations to seven categories is shown in Figure 1. Additional file 2 lists all duplications and inversion structural variations, not further discussed herein. Altogether, we observed an average of 14.4 ± 6.8 polymorphic variations of all types per ~930 bp open reading frame, similar to what we found in OR pseudogenes (14.9 ± 6.7, p = 0.0881 using Kolmogorov-Smirnov test). The combinations of polymorphic variations within each OR open reading frame are subsequently used to define haplotypic OR alleles at the DNA and protein levels (see below). All variations are available at the Human Olfactory Receptor Data Explorer database (HORDE database, http://genome.weizmann.ac.il/horde/) [6,44,45].

**Figure 2:**
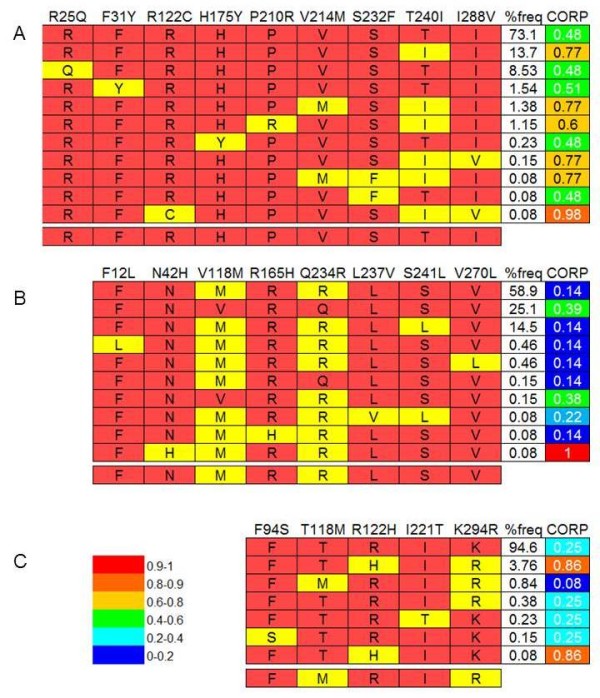
**OR protein haplotype alleles for selected ORs.** This is shown for OR1D2 (**A**), OR4E2 (**B**) and OR7C2 (**C**), typifying genes with high inter-allele diversity of CORP-predicted functionality. Segregating protein positions (indicated on top) are shown for each haplotype sequence, with yellow indicating non-reference SNP allele. The ancestral chimpanzee allele is shown in the lower row of each panel. The frequency of each allele in the population (%freq) and the CORP pseudogene probability score [43] are indicated in the two right columns. A high CORP score predicts a high pseudogene probability.

We subsequently analyzed 2610 missense variants found in the imputed and haplotype-phased data of the 1000 Genomes Project for 651 individuals, to obtain 4069 putative haplotypic OR alleles. Of these, 2682 alleles are present in 3 or more individuals, and hence are less likely to be false positives (Additional file 3). A display of allelic diversity for 30 typical OR loci indicate as many as 35 haplotypic proteins per locus, with an average of 10.4 ± 6.7 (Additional file 1: Figure S1). Every one of these allelic DNA sequence variants ostensibly represents a distinct functional protein, portrayed by a color-coded functional score based on the previously published CORP algorithm [43], including indications for probable non-functionality (CORP>0.9). Figure 2 shows three OR genes with maximal CORP score inter-allele diversity. We also portray three genes with reported odorant specificity [15,39,46]. For the androstenone-binding OR4D7, all 8 haplotypic alleles have similarly high degree of predicted functionality. For the aliphatic thiol-specific OR2C1 the 11 alleles have similar intermediate-level functionality prediction. In contrast, for the amyl butyrate-binding OR2AG1 a bimodal distribution of predicted functionalities is seen, pointing to the possibility of modified odorant responses (Additional file 1: Figure S2).

**Figure 3:**
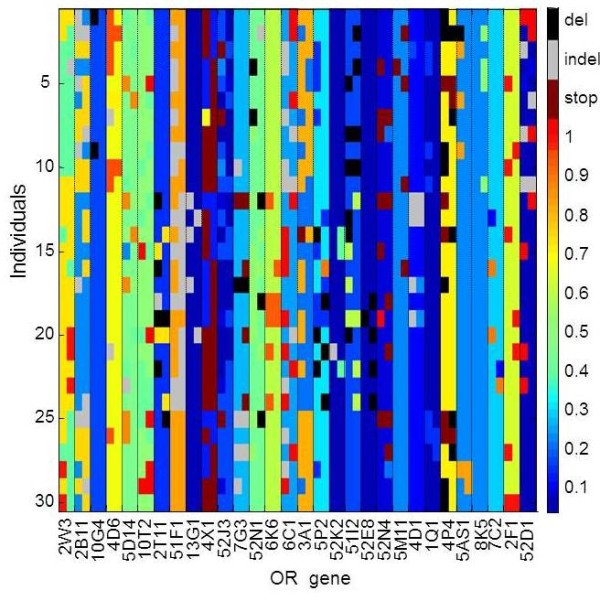
**Protein allele genotype for 30 selected OR genes in 30 individuals.** The ORs and individuals were selected to show maximal inter-allele diversity of CORP-predicted functionality. The two allelic protein sequences at each locus are shown, color-coded by their CORP scores for missense, and as indicated by the abbreviations (see Figure 1) for nonfunctional, and as depicted by the color scale on right, Ethnicities: 1–11 Europeans, 12–26 Africans, and 27–30 Asians.

**Figure 4:**
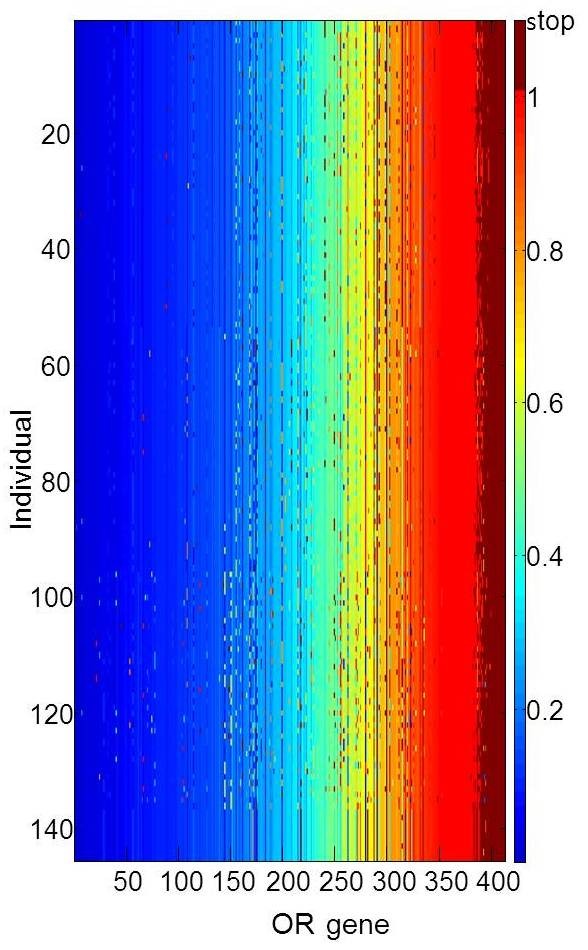
**Genomic variation for the entire OR intact haplotype repertoire in 145 individuals.** Every individual is represented in every locus by a single randomly selected missense allele, except for Stop loci for which the non-reference allele is preferably shown. Color coding as in Figure 3. Ethnicities: 1–53 - Asians, 54–95 - Europeans and rows 96–145 - Africans. The dataset does not include alleles with concomitant indels and CNV deletions.

Figure 3 shows a variation matrix for the 30 OR loci, selected for showing maximal diversity of CORP score values, as viewed in a subset of 30 representative individuals carrying such genotypes. A summary of such patterns for all 413 intact ORs and in 145 individuals of the three major ethnic origins (Figure 4) highlight the vast inter-individual variation in this chemosensory receptor system.

**Figure 5:**
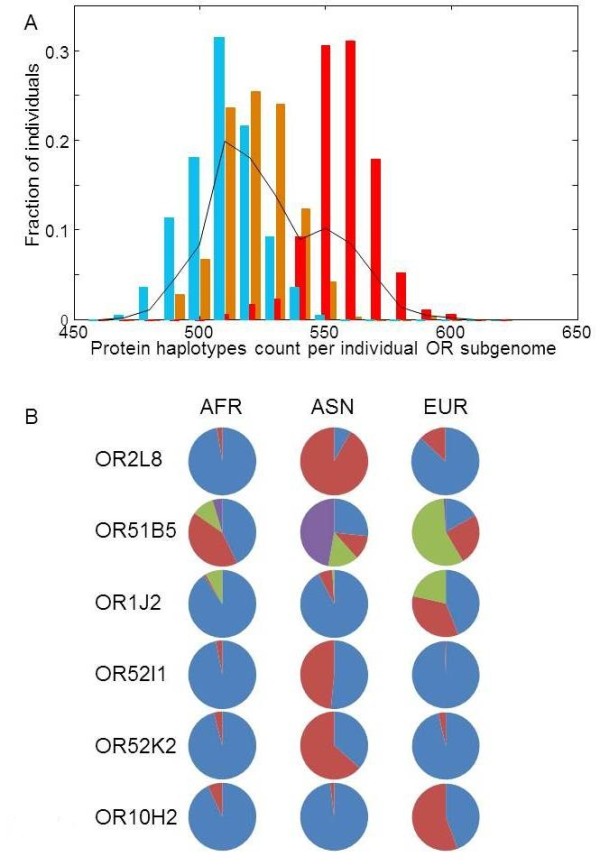
**Population differences of personal OR protein allele counts.A**) Distribution of the OR missense allele count frequencies in Africans (red), Europeans (brown) and Asians (blue). The black line indicates the average distribution for the whole population. **B**) Haplotype allele frequencies for six OR genes that show the highest inter-population variability. Only alleles with 1000 Genomes frequency > 10% in the entire human population are shown. AFR- Africans, ASN- Asians, EUR- Europeans.

The foregoing analysis embodies a significant enhancement of the OR repertoire in every human individual via haplotypic diversity. Thus, a large majority of human individuals analyzed harbor 490–570 different haplotypes at the 413 loci, i.e. 85–165 loci in a heterozygous state (Figure 5A). This amounts to a repertoire augmentation of 20–40%. The three ethnic groups have pronouncedly different allele count distributions, with Africans having an especially high average of 557 ± 13 different OR sequence variants per individual (Figure 5A). Different ORs often have dissimilar variant distribution in the three populations as exemplified in Figure 5B. These results are consistent with the idea of African origin of modern humans [47,48].

### Nonfunctional variations

**Figure 6:**
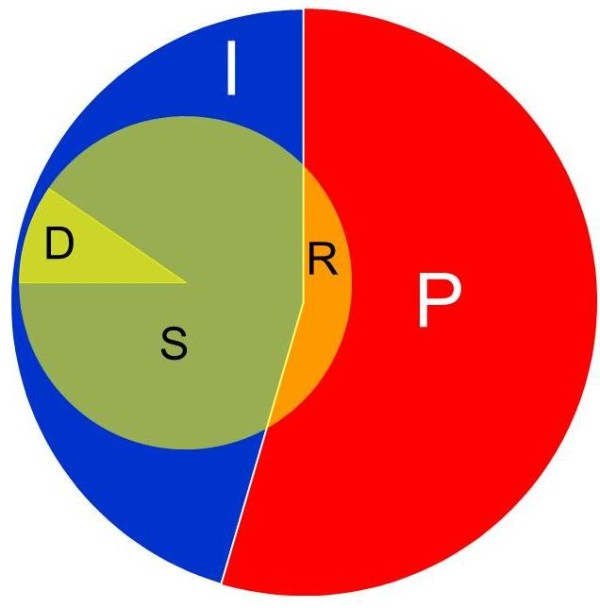
**A status diagram of the human OR repertoire.** Among the 851 human OR sequences in the reference human genome, 464 were originally annotated as pseudogenes (**P**) and 387 as intact genes (**I**). Our study suggests that 218 (56%) of these intact ORs are segregating pseudogenes (**S**, originating from stop-SNPs and frame-disrupting indels), and 27 (6.9%) have a CNV deletion allele (**D**). Additionally, 26 (5.6%) of the OR pseudogenes are “resurrected (**R**)”, by showing an intact allele in some individuals.

We next focused on the analysis of nonfunctional variations that eliminate specific members of the OR allele repertoire in a given person, hence are excellent candidates for underlying inter-individual odorant threshold differences [18,49]. First, we analyzed small events, i.e. stop SNPs and indels (up to 84 bases) that result in frame disruption, as derived from 6 different data sources (Additional file 1: Table S1 and Figure S3). Among the 387 OR loci annotated as intact genes in the reference genome we identified 218 cases for which at least one nonfunctional allele was seen. In addition, among the 464 ORs defined as pseudogene in the reference genome, we identified 26 ORs that harbor an intact allele in at least one person, and may be considered as “resurrected” from fixed pseudogene status (Additional file 4). Thus, among 413 thus defined intact loci, a total of 244 loci (59%) show segregation between intact and nonfunctional alleles (segregating pseudogenes, Figure 6). This provides a major enhancement relative to our previously published set of 31 segregating pseudogenes [25]. When analyzing 145 subjects from the 1000 Genome Project for which both SNPs and indels are available, we found that every human individual has 21 ± 4 deletion heterozygotes and 11 ± 2 loci that are homozygously disrupted.

We performed experimental validation for 68 nonfunctional SNPs (stop gain, stop loss, and loss of initiator methionine) and 200 frame-disrupting indels (Additional file 4). For this we designed a custom SNP array (Illumina GoldenGate) that included the total of 268 nonfunctional variations. These were genotyped in a cohort of 468 individuals of two ethnicities, providing validation for 184 of the variations, as compared to a most probable value of validation of 197 ± 2 based on the cohort size and specific minor allele frequencies (validation rate of 93.4%). The number of nonfunctional SNPs per individual (heterozygous and homozygous) thus discovered is shown in Additional file 1: Figure S4. A significant correlation was seen between the allele frequencies in the 1000 Genomes Project data and our validation sets (Additional file 1: Figure S5).

### Deletion CNVs

**Figure 7:**
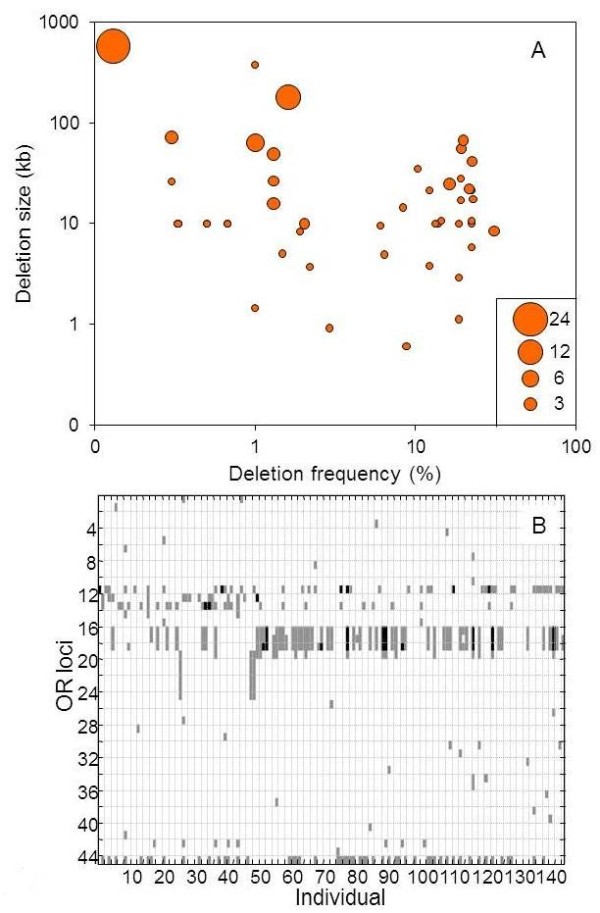
**Deletion CNV events in the human OR repertoire.A**) The deletions size plotted against the deletion frequency in the 145 individuals analyzed. Circle size represents the number of OR genes affected by the deletion. **B**) Genotype calls for the 45 biallelic deletion loci [30] in 145 individuals. Black, homozygotes; grey, heterozygotes.

We further performed integration of biallelic deletion CNVs for all OR loci, utilizing five different data sources (Additional file 1: Table S1). This revealed 63 such CNV events (Figure 7A, Additional file 5). This brings the total number of loci that harbor a nonfunctional allele in the examined populations to 271 (Figure 6). As previously seen for segregating pseudogenes [26], here too we observe a great inter-individual variation in the combinations of OR loci affected by deletion CNVs (Figure 7B).

**Figure 8:**
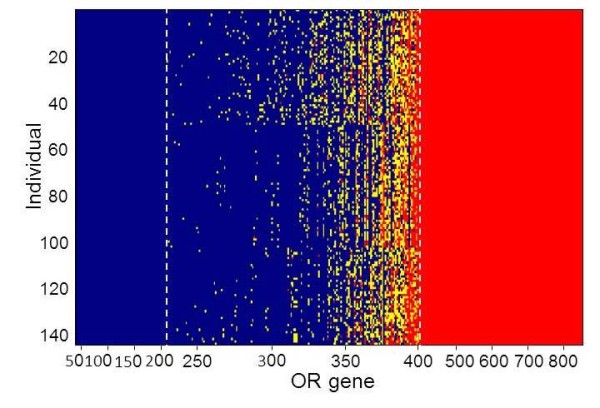
**Personalized OR repertoires in 145 individuals.** Blue- homozygotes for an intact allele, red- homozygotes for a disrupted allele, yellow- heterozygotes. Nonfunctional allele calls: stop SNPs, indels and deletion alleles.

**Figure 9:**
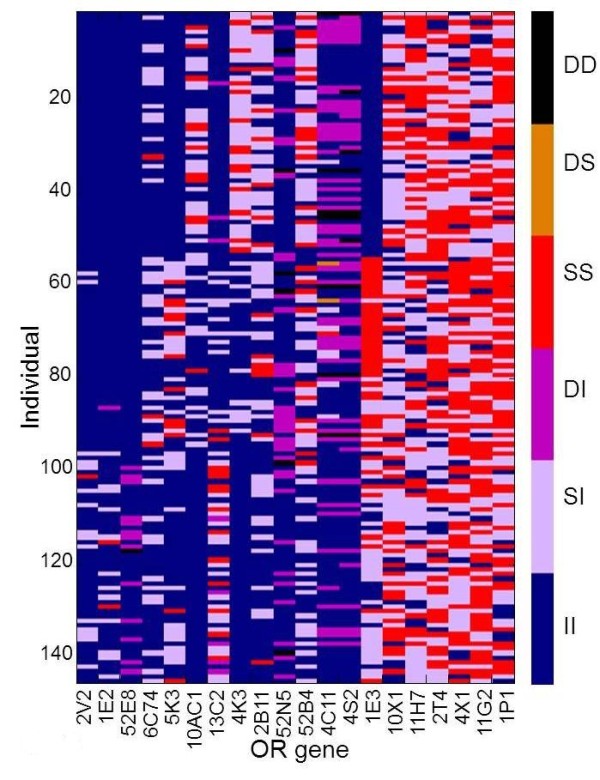
**Nonfunctional allele genotypes for 20 OR genes in 145 individuals.** The genes have been selected to maximally span the genotype range. Individuals are sorted by ethnicity as in Figure 4. Allele statuses are: intact (I), nonfunctional SNPs/indels (S), bi-allelic deletion CNV (D) [30]. Colors indicate genotypic combinations. The full matrix with all 177 ORs in 145 individuals is shown in Additional file 1: Figure S7.

**Figure 10:**
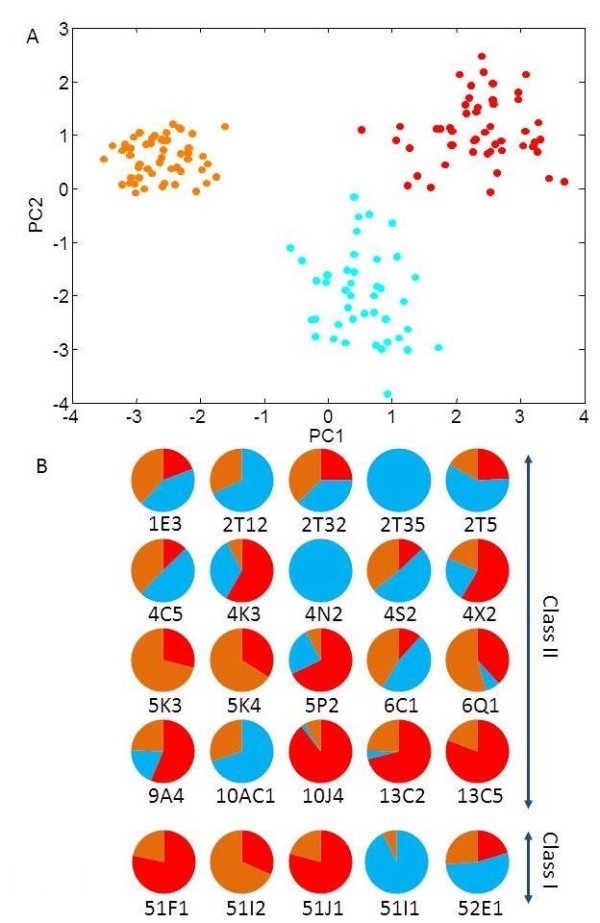
**Population differences of OR SPGs.A**) Principal component analysis of the nonfunctional SNP genotypes. Each point represents a specific individual, colors as in Figure 5A. **B**) Normalized relative frequencies of the nonfunctional OR allele in the three ethnic populations, color-coded as in (**A**). This is shown for 25 ORs, selected to represent the highest inter-population variability (values are given in Additional file 5). This include 20 ORs belonging to class II (“tetrapod-like”), members of 15 subfamilies (e.g. 1E), and 5 ORs belonging to class I (“fish-like”), represented by members of 5 subfamilies (e.g. 51F). OR classification is as described [3]. Colors as in Figure 5A.

The combined variation results of the deletion CNVs with the SPG genotypes strongly reinforce the notion that practically every individual in the human population has a different combination of intact and inactive alleles (Figure 8). Using a phasing procedure (see methods), we could assign deletion locus haplotypes to 177 ORs, which in some cases harbor more than one event on a given chromosome, and in others create compound heterozygosity for two deletion types (Figure 9 and Additional file 1: Figure S6). Using this combined view we find that, on average, every individual genome carries a disrupted allele at 35 ± 4 loci, of which 11 ± 3 are homozygously affected (Additional file 1: Figure S7). Because every olfactory sensory neuron expresses a single allele at an OR locus, heterozygosly deleted SPGs might have a phenotypic outcome. The personalized repertoire of intact and inactivated ORs significantly differs among ethnic groups (Figure 10A), and such differences are dominated by a subset of OR loci, representing both class I and class II ORs, that manifest a relatively large inter-group variation (Figure 10B, Additional file 1: Table S2). There is however no significant difference in homozygous deletion alleles among the different populations (Additional file 1: Figure S6).

### OR Evolution

**Figure 11:**
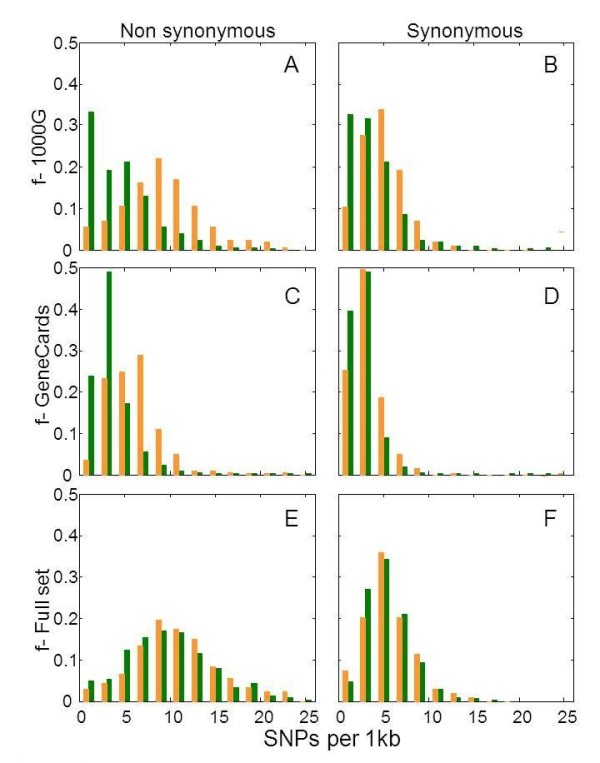
**OR genes are enriched with non-synonymous SNPs.** Each panel compares the frequency distribution (**f**) for ORs (orange) and control genes (green). The analysis is done using three data sources: 1000 Genomes Project (**A, B**) with 581 genes with a single coding exon as controls; the GeneCards database [50] (**C, D**), with 15,425 protein coding genes as controls; all 10 data sources (Additional file 1: Table S1) (**E, F**), with OR pseudogenes as controls. A, C, E, non-synonymous SNPs, B, D, F, synonymous SNPs.

We asked whether OR genes harbor an unusually high frequency of missense variations. For this, we compared the number of non-synonymous SNPs in two gene sets. The first was 387 OR genes defined as intact in the reference genome, and the second control set constituted 581 protein-coding genes that (like ORs) have a single coding exon. The latter included non-OR G-protein coupled receptors, keratin associated proteins, protocadherins and histones. ORs were found to have 7.7 ± 4.3 missense SNPs per open reading frame, while the controls had 2.2 times less such SNPs (3.5 ± 4.3, p<2.2X10^-16^ Wilcoxon rank sum test with continuity correction, Figure 11A). This was confirmed in a second test set of 15,425 protein coding genes (all GeneCards coding SNPs [50,51] Figure 11C, p<2.2X10^-16^ ). Synonymous SNP counts showed a much smaller, though significant, difference between ORs and controls (Figure 11B, p = 1.465X10^-13^ and Figure 11D, p = 0.00789). We note that OR genes and pseudogenes show a similar propensity of non-synonymous SNPs (Figure 11E), with a slight, statistically significant excess in intact ORs (p = 0.001149). The simplest interpretation is that on average ORs may neutrally accumulate genetic variations, mainly due to less stringent purifying selection as compared to non-ORs [31,32].

**Figure 12:**
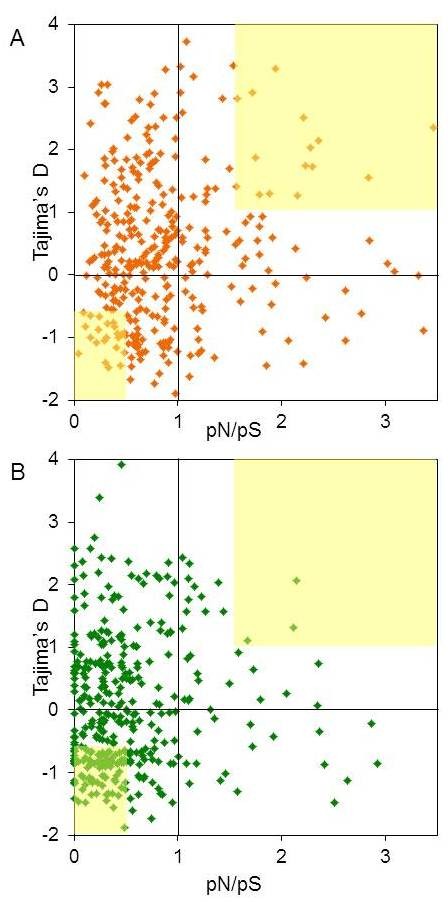
**Selection signatures in the OR genes.** Correlation of non-synonymous to synonymous substitution rate (pN/pS) with Tajima’s D values for A, 364 intact OR genes and B, 439 single coding exon genes. Data are plotted for the European population, other populations in Additional file 1: Figure S4. The difference between the ORs and the control genes was tested using the Kolmogorov-Smirnov test yielding p = 1.936*10^-24^ for pN/pS, and p =2.26*10^-5^ for Tajima’s D. The yellow squares highlight regions which might act under non-neutral selection, top right with D>1, pN/pS>1.5 (balancing selection), and bottom left with Tajima’s D<−0.5 and pN/pS<0.5 (purifying selection). Additional file 1: Table S3 lists the 57 ORs found under purifying selection in all populations.

We asked whether some of the OR genes accrue variations in a non-neutral fashion by examining the ratio of polymorphic non-synonymous substitutions per non-synonymous site to polymorphic synonymous substitutions per synonymous site (pN/pS) [52,53], whereby a value near one would suggest neutrality. While for most ORs the results are consistent with neutrality, there is significant enrichment in the high pN/pS region of the distribution in ORs compared to controls, consistent with selection (Figure 12 and Additional file 1: Figure S8). A subclass of the ORs with pN/pS>1.5 also have a positive value of Tajima's D (Figure 12A) suggesting balancing selection. We asked whether the subgroup of fast evolving ORs (with pN/pS>1.5) is enriched with “evolutionary young” genes, defined as those lacking one-to-one orthology relationships with the chimpanzee orthologs [29]. We find that no such enrichment occurs, as among 47 fast evolving ORs, the fraction of evolutionary young genes is 12.8%, while for all other ORs the fraction is 17.1%. We further note that a relatively small subgroup of 57 ORs (16.8%) in our dataset (in all three populations) show evidence for strong purifying selection (Tajima’s D<−0.5 and pN/pS <0.5, Figure 12). This low count as compared to 40.5% in controls, is likely related to the tendency of ORs to evolve towards higher inter-individual diversity [54]. Thus, for the specific receptors showing this evolutionary pattern (Additional file 1: Table S3), such sequence conservation may indicate functional importance, e.g. recognition of essential odorants essential for the species as a whole.

## Discussion

### An OR variation compendium

Using various databases and experimental resources, we have compiled a compendium of synonymous, missense and nonsense SNPs, as well as copy number variations within OR coding regions. A major resource for this work was the 1000 Genomes Project’s whole genome sequence data [55], yielding variation and phase information. A significant caveat regarding such data is their low coverage in each sequenced individual and the imputation procedures used in the phasing process [56-58]. This is partly ameliorated by the fact that the main body of our analyses is based on cumulative data from 300–1300 human chromosomes. Another point of concern is that some of the variations were obtained from dbSNP [59], for which population frequencies or validation are sometimes not provided. Indeed, in our experimental validation of 268 OR nonfunctional SNPs, a majority (65%) of the unsupported variations were mined only from dbSNP.

### Enormous gene variability

Our results portray an overview of the degree of inter-individual genomic variability harbored in the OR gene inventory. We report on an enormous amount of genomic variation (one variation per 66 bases), 2.5 times larger than in single coding exon control genes. Our analyses suggest that such enhanced variation is largely due to neutral drift, both because the propensity of variations per coding region is similar to that found for OR pseudogenes, and since the average pN/pS value for the intact ORs is 0.9 ± 0.6, consistent with neutrality.

Previous studies reported on positive selection acting in specific OR genes [60-62], potentially related to a recent evolutionary acquisition of a capacity to recognize specific behavior-related odorants [63]. Our results do not provide clear evidence for such selection mode. Other reports suggest that the OR diversity may be maintained to some degree by balancing selection [54,64], similar to that acting upon the major histocompatibility complex alleles [65,66], leading to enhanced ligand recognition success at the population level [67]. While balancing selection for ORs has been disputed [68] our results suggest that a fraction of OR genes may be under such selection mode, a mechanism consistent with the advantage for heterozygosity in a pathway endowed with allelically excluded expression. This is in line with a previous report showing higher than expected count of heterozygotes at OR SNPs in the HapMap populations, which led to the conclusion that the human ORs may have been shaped by balancing selection, stemming from overdominance [54].

Weak purifying selection has also been suggested to affect a subpopulation of human ORs, as seen by human-chimpanzee comparisons [69]. In line with this, we identified nearly 60 ORs in our dataset showing evidence for this evolutionary mechanism. Such evolutionarily conserved OR genes may subserve the recognition of specific odorants important for survival and/or propagation of the species. Interestingly, this group of human genes has a higher fraction of candidate orthologs in mouse, as compared to dog, consistent with a presently accepted phylogeny whereby primates and rodents belong to the same clade, different from that of carnivores [70,71], although a rodent-outside phylogeny was also suggested [72,73].

In sum, it is difficult to negate the possibility that certain modes of selection act on subsets of human OR genes, but it is rather certain that no single mode applies to all ORs. Such heterogeneity of selection modes within the large OR repertoire has also been reported in dog [74,75].

### The human allele repertoire

Irrespective of evolutionary path, it is obvious that human ORs show an unusually high variability as compared to other intact protein-coding genes. We report that some human individuals have as many as 600 OR coding regions at their ~400 intact OR loci. Some of these allelic protein variants may have different odorant affinity and/or specificity [39]. Previous reports demonstrate that olfactory sensory neurons express only one of the two alleles at a given locus [2,76,77] with a possibility that allelically excluded neurons report independently to olfactory bulb glomeruli in the brain [78]. This, together with allele plurality, generates a powerful mechanism for augmenting functional variation and enhancing odorant recognition capacities. Furthermore, a higher size of the effective OR repertoire may also signify enhanced average sensitivity to odorants [79,80]. The functional significance of allelic diversity most likely applies to other species as well [75,81].

### Loss of function alleles

One of the striking results of the present report is the extremely high prevalence of loss-of-function OR alleles. Based on the data mining performed, among the 851 human genomic OR loci, 438 have a frame-disrupting pseudogene apparently fixed in the entire population. Of the 413 remaining loci, 271 (66%) have at least one allele lacking an intact open reading frame, including frame disruptions and deletion CNV alleles. The CORP algorithm [43] predicts that an additional 37 loci have missense nonfunctional alleles, with a CORP score > 0.9, suggesting a probable non-functional OR protein. Thus, as many as 308 OR loci harbor one or more functionally disrupted alleles, and only 105 loci appear to be purely functional in the studied population. This is likely related to the emergence of a large number of OR pseudogenes in higher primate evolution [22,82]. Further, the very high incidence of segregation between intact and nonfunctional alleles attests to a possible highly accelerated gene inactivation in recent human evolution. This potentially took place on a shorter time scale than the previously indicated human-specific acceleration in OR pseudogene accumulation relative to apes [24].

The presently reported number of 308 non-intact loci is fivefold larger than an earlier estimate of ~60 [26]. This number will likely increase even further as many more human genomes become available. Curiously, among the non-intact loci are included 26 that were originally annotated as pseudogenes in the reference genome. Further sequencing would probably show additional such cases of “resurrected” ORs, most likely from among the 44 fixed OR pseudogenes that have only one frame disruption [6,45]. It should be pointed out that OR pseudogenes are not processed pseudogenes [83], and hence are typically endowed with all features of intact ORs (cis regulatory elements, 5’ upstream introns and non-coding exons) and are only different from the intact form by frame-disrupting mutations.

### Personal noses

**Figure 13:**
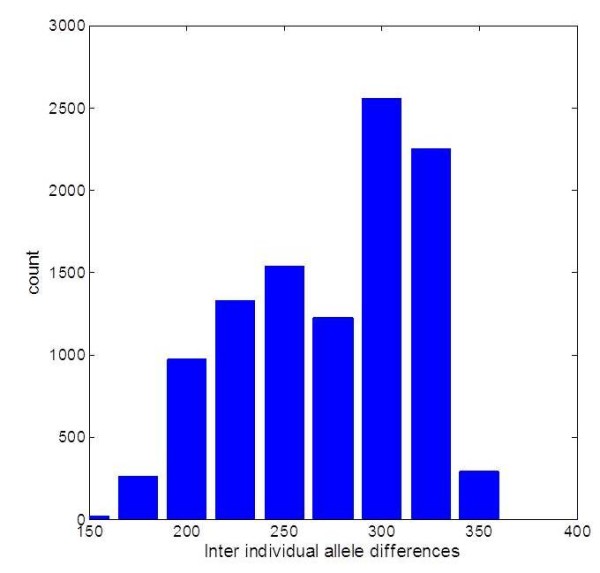
A distribution of the pairwise inter-individual OR missense allele count differences for all 145 individuals shown in the previous figures.

Our comprehensive portrayal of genetic variability in OR genes provides considerably enhanced support for the notion of “different noses for different people” [26]. While for the 145 individuals analyzed from the 1000 Genomes Project data the overall count of homozygous deletion genotypes per individual is not very high (16 ± 3 including missense nonfunctional alleles), the inter-individual variability is vast: there was no case of two individuals having the same deletion pattern across all relevant loci. Furthermore, viewing the broader picture of nonfunctional alleles of all types, as well as protein missense alleles, a randomly selected pair of subjects will on average share only 500 of the alleles, and the remaining 274 (33%) will be different (Figure 13). Importantly, on average 32% of all fully intact OR loci are heterozygously disposed, encoding two different active OR protein variants. A heterozygous deletion event affecting such a locus could have an odorant sensitivity phenotype, as only one of the two different functional alleles would remain active, and the allelically-excluded neuronal pattern could thus be modified.

Analysis of deletion CNVs with high-confidence breakpoints revealed that, for a typical individual, 40% of the deletion CNVs affect more than one (and up to six) intact OR genes, consistent with previous reports [29,30], thus highlighting the large impact of CNVs as opposed to smaller variants. However the contribution of deletion CNVs to the overall number of disrupted alleles per individual is less pronounced.

### Receptor diversity and ethnogeography

Our results generally suggest substantial differences among the three major ethnogeographical groups analyzed: Caucasians, Africans and Asians. The most significant result is that Africans have a higher number of OR protein haplotypic variants, with implications to chemosensory diversity. Such findings are in line with the reported higher genetic diversity in this ethnogeographical group [48,84,85]. Some of the protein variants are seen only in one or two of the groups, and others show great disparity of relative allele frequency. The three different human races also have distinct patterns of deletion allele genotypes, which again could affect chemosensory preferences. Previously, we have reported a slightly higher number of intact OR loci in Africans as compared to Caucasians [26]. The results reported here, utilizing a much larger number of deletion loci, shows no statistically significant difference in this realm between ethnic groups.

## Conclusions

We used data mining strategies to generate a comprehensive compendium of genomic variations in the inventory of human OR coding regions. Our analyses suggest that the effective size of the functional human OR repertoire is much higher than the number of intact loci, implying considerable enhancement of the potential of human smell perception diversity. Importantly, using both data-mining and experimental verification we show that more than two thirds of human OR loci segregate between an intact and inactivated alleles. These results portray a case of unusually high genetic diversity, and suggest that individual humans have a highly personalized “barcodes” of functional olfactory receptors, a conclusion that likely applies to other receptor multigene families as well.

## Methods

### Genomic variations

Table S1 (Additional file 1) lists the data sources screened for genomic variations in the OR coding regions [26,30,55,59,86-93]. We used the UCSC table browser tool [94] to extract variations from dbSNP, and custom Perl scripts for other databases. We used the GRCh37/hg19 reference genome assembly, and when necessary genomic variations were converted to this version, using the liftOver tool (http://genome.ucsc.edu/cgi-bin/hgLiftOver). Variations that had the same type (SNP or CNV) in the same OR gene symbol with the same start and end locations were considered duplicates and were merged. Indel variations, often located in oligonucleotide repeat loci [95], might have more than a single valid mapping, and were therefore merged manually. Annotation and classification of the variations into the different categories presented in Figure 1 was done by a custom Perl script. Multi-allelic SNPs were removed from the analysis. Unique genomic mapping for dbSNP variations was ascertained by allowing only SNPs with “map weight” equal to 1. SNPs from other sources were analyzed for non-uniqueness by mapping flanking sequences (±50pb) with BLAT [96] and filtering out cases with multiple locations with ≤2 mismatches.

Bi-allelic CNV deletions reported by different sources (Additional file 1: Table S1) were merged by the following procedure: if both beginning and end coordinates of two CNV instances differed by ≤1 kb they were merged into a single entry, and the average genomic coordinates and allele frequencies were used (Additional file 4). From the 1000 Genomes Project data for the first 150 individuals ([93], union.2010_06.deletions.sites.vcf) we kept only deletions with allele frequencies. Multiple overlapped variants from this source were filtered using the following rules (in order):i) When a deletion spanning multiple ORs overlapped with deletions of individual ORs in the same location, the former was preferred; ii) Among overlapping deletions affecting the same OR, the smallest was favored.

OR haplotypes were computed based on phased SNP calling data from the Broad Institute Phase 1 1000 Genomes Project data files (http://www.1000genomes.org/) (AFR.BI_withr2.20100804.genotypes, ASN.BI_withr2.20100804.genotypes, EUR.BI_withr2.20100804.genotypes). Each OR haplotype was defined as a binary vector of non-synonymous segregating sites present in all 3 populations, with 1 denoting the non-reference variant. The OR haplotype frequencies for each population were then summarized in Additional file 3.

### Haplotype protein functional score

The CORP routine, available in the HORDE database (http://genome.weizmann.ac.il/horde/)[43,45], was used to assign a functional score for each haplotype. CORP examines the amino-acid composition of 60 highly conserved pre-defined positions, where for each site a specific list of present amino-acids is defined. Using a logistic regression model, CORP score (*CS*) is computed using:

(1) $$CS=\frac{1}{1+\exp\left(S\right)}$$

where *S* is a weighted sum of β coefficients [43]

(2) $$S=-50+\sum_{i=1}^{60}\alpha_{i}\times \beta_{i}$$

and *α*_*i*_ = −1 if in the sequence carries an allowed amino-acids in position *i*, and *α*_*i*_ = 1 otherwise.

### Variation frequency comparisons

Two control sets were used for variation frequency comparisons. The first was 581 single coding exon genes, retrieved from GeneLoc ([97], http://genecards.weizmann.ac.il/geneloc), further curated with the UCSC table tool [94] to remove non-protein-coding genes. SNPs in these genes were extracted from the 1000 Genomes Project data for the same set of 651 individuals and using the same computational procedures as applied to the ORs. The SNP count was normalized to gene length using the longest transcript.

The second control gene set was of 15,425 protein coding genes, extracted from GeneCards (http://www.genecards.org/, [50,51]). The same source was also used to obtain SNPs in the 321 intact ORs listed within it. SNPs in OR pseudogenes were classified as “synonymous” or “non-synonymous” based on sequence translation using FASTY [98]. For calling reversion of a pseudogene to an intact status, an open reading frame ≥300 amino-acids was used as a cutoff.

### DNA samples

For SNP validation, a cohort of 480 DNA samples was used, collected under ethically-approved protocols as described [91,99]. This panel included 366 individuals of Israeli Jewish origin (271 Ashkenazi, and others of mixed origin) used in a previous study [99], as well as 92 individuals of American origin (57 Caucasians and 22 Afro-Americans) was collected in the framework of a collaborative genotype-phenotype study [91,100].

### SNP genotyping

Genomic DNA was extracted from 10 ml of peripheral blood using a DNA Isolation Kit for Mammalian Blood (Roche) [99]. DNA concentration was measured in the Beckman DTX880 Multi-Detection Microplate Reader using PicoGreen (Invitrogen). Genotyping of SNPs was carried out at the Rappaport Research Institute, Technion, Israel, using the Illumina GoldenGate assay according to the manufacturer’s instructions (Illumina Inc., SanDiego, CA, USA) http://www.illumina.com/technology/goldengate_genotyping_assay.ilmn.

The Illumina oligonucleotide pool assay (OPA) was designed using the Illumina Assay Design Tool (ADT) software, with inclusion of all OR nonfunctional variations showing an ADT designability score > 0.4. Inter-variation distances were kept at >60 bp, choosing the variants with highest designability score. The final design included 285 nonfunctional OR variations, of which 268 were successfully genotyped.

For computing the most probable value of validation, we used the minor allele frequencies for the genotyped SNPs, as shown in Additional file 1: Figure S9. We simulated 1000 cohorts of 445 individuals (to account for averaged null calls of 22 individuals per SNP) and obtained a mean and standard deviation for the rate of validation for each variant.

### Resolving genotype ambiguities

We developed procedures to obtain unambiguous personal genotypes based on the mining of three independent genotype datasets: 1) The 1000 Genome Project imputed phased SNPs (Broad Institute, version 20100804); 2) The 1000 Genome Project imputed phased indels (Broad Institute, version 2010_07); 3) Bi-allelic CNV calls as described [30]. Ambiguities arise when more than one of these sources reports heterozygosity in the same person and in the same gene. Regarding the merger of nonfunctional SNPs with indels, only 3 genes (OR1B1, OR4C5, OR7G3) showed such an ambiguity, and it was resolved by re-phasing using the PHASE program [101]. The merger of CNV deletions with SNPs/indels was done by the following rules: a. for homozygous CNV deletion concomitant with nonfunctional SNP/indel, the latter was considered as imputation artifact and was ignored; b. heterozygous CNV deletion concomitant with apparently homozygous nonfunctional SNP/indel, was scored as compound heterozygosity; c. Heterozygous SNP/indels along with heterozygous CNV remained unsolved (3 cases). For Figure 3, in cases of unresolvable heterozygous indel/deletion along with claimed missense heterozygosity, one missense allele was selected randomly.

### Analyses of selection signatures

The ratio of the number of polymorphic non-synonymous substitutions per non-synonymous sites to the number of polymorphic synonymous substitutions per synonymous sites (pN/pS) was calculated for ORs and control genes following published procedure [102] and using SNPs of the 1000 Genomes Project. This procedure was demonstrated to be correlated with Ka/Ks for divergence [102]. Tajima’s D Neutrality test was computed with the DnaSP program [103].

## Abbreviations

OR, Olfactory receptor; SPG, Segregating pseudogene; CNV, Copy number variation; SNP, Single nucleotide polymorphism; CORP, Classifier for Olfactory Receptor Pseudogenes; pN/pS, The ratio of polymorphic non-synonymous substitutions per non-synonymous site to polymorphic synonymous substitutions per synonymous site.

## Competing interests

The authors declare that they have no competing interests.

## Authors’ contributions

TO, SW and DG performed the computational mining of genomic variations. TO, SW, MV and AR analyzed the data. TO, DL and SW wrote the paper. CJW, MK and EBA did the experimental validation. NN worked on the database design. All authors read and approved the final manuscript.

## Supplementary Material

Additional file 1 Figures S1-S9, Table S1, Table S2, Table S3. Click here for file

Additional file 2 A List of duplications and inversions in the OR genes. Click here for file

Additional file 3 **OR protein haplotypes.** Haplotypes are represented by their segregating positions (fourth column) where 0 is reference-genome allele and 1 is non-reference allele. Segregating position names are composed from the chromosome name, genomic coordinate, reference amino-acid, protein position and non-reference amino-acid. Click here for file

Additional file 4 A list of nonfunctional variations in the OR genes. Click here for file

Additional file 5: OR intact loci for with bi-allelic deletion allele. Click here for file
