# Supplementary material for: Personal receptor repertoires: olfaction as a model
Source: BMC Genomics. 2012 Aug 21;13:414. doi: 10.1186/1471-2164-13-414 (PMC3462693; doi:10.1186/1471-2164-13-414)
Supplement: Additional file 1 — Figures S1-S9, Table S1, Table S2, Table S3. [file 1471-2164-13-414-S1.pdf]

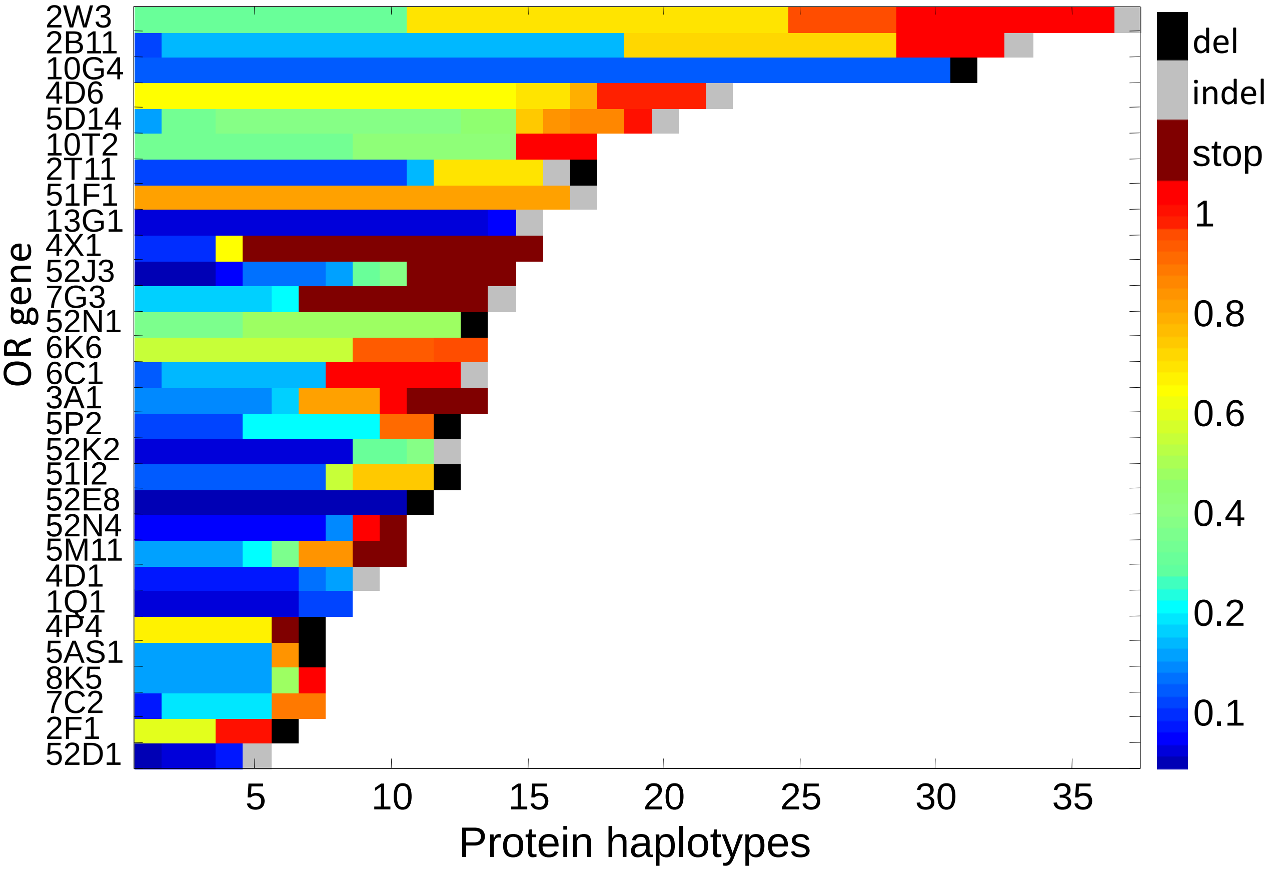

Figure S1: Protein haplotypes of the ORs shown in Figure 3. The color coding is the same.

OR7D4 (Androstenone [39])

P79L	S84N	R88W	T133M	G227R	K232E	%freq	CORP
P	S	R	T	G	K	79.19	0.08
P	S	W	M	G	K	16.44	0.08
L	S	R	T	G	K	2.84	0.19
P	N	R	T	G	K	1.15	0.08
P	S	R	T	R	K	0.15	0.08
P	S	R	T	G	E	0.08	0.08
L	S	W	T	G	K	0.08	0.19
P	S	R	M	G	K	0.08	0.08
P	N	R	T	R	E		

OR2C1 (Octanethiol, Nonanethiol [15])

G16S	P58S	C169Y	R172W	E180K	R229H	P262L	A300T	G312D	%freq	CORP
G	P	C	R	E	R	P	A	G	70.28	0.56
S	P	C	R	E	H	P	A	G	24.19	0.56
G	S	C	R	E	R	P	A	G	2.07	0.73
S	P	C	R	E	H	P	A	G	1.31	0.56
G	P	C	R	E	R	P	A	D	0.84	0.56
G	P	C	R	E	R	P	T	G	0.69	0.56
G	P	C	W	E	R	P	A	G	0.15	0.56
G	P	C	R	E	R	L	A	G	0.15	0.56
G	P	C	R	E	H	P	A	G	0.15	0.56
S	P	C	R	K	H	P	A	G	0.08	0.56
G	P	C	R	K	R	P	A	G	0.08	0.56

no chimpanzee ortholog

OR2AG1 (Amylbutyrate [46])

N42S	Q65H	L114P	L135V	C141S	H183P	V187L	I221N	P262T	%freq	CORP
N	Q	L	L	C	H	V	I	P	72.81	0.12
N	Q	L	L	C	H	L	I	P	20.74	0.12
S	Q	P	L	C	H	V	I	P	2.15	0.99
N	Q	L	V	C	H	L	I	P	1.61	0.12
S	Q	P	L	C	H	V	I	T	1.08	0.99
S	H	P	L	C	H	V	I	P	0.46	0.99
N	Q	L	L	S	H	V	I	P	0.46	0.12
S	Q	L	L	C	H	V	I	P	0.23	0.99
N	Q	L	L	C	H	V	N	P	0.23	0.12
N	Q	P	L	C	H	L	I	P	0.15	0.27
N	Q	L	L	C	H	L	I	P	0.08	0.12
N	Q	L	L	C	H	L	I	P		

0.9-1.0
0.8-0.9
0.6-0.8
0.4-0.6
0.2-0.4
0-0.2

Figure S2: OR protein haplotype alleles for three OR genes with reported odorant specificity, as indicated in parenthesis.

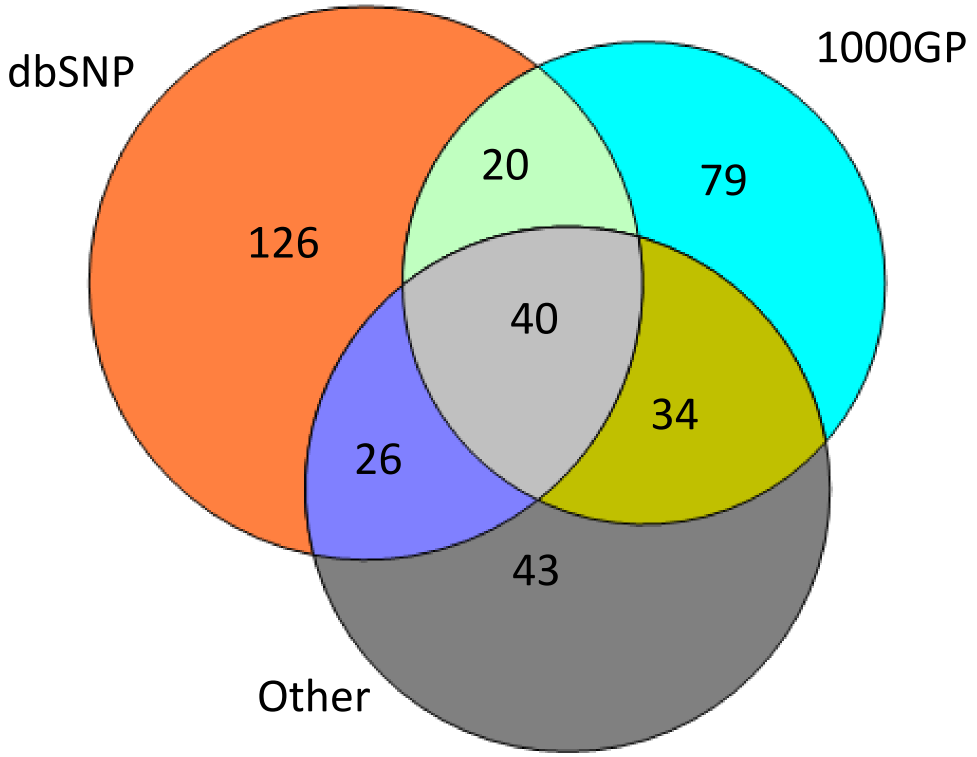

Figure S3: Venn diagram of the deleterious SNPs identified from dbSNP, the 1000 Genomes Project (1000GP) and other sources listed in Table S1 (other).

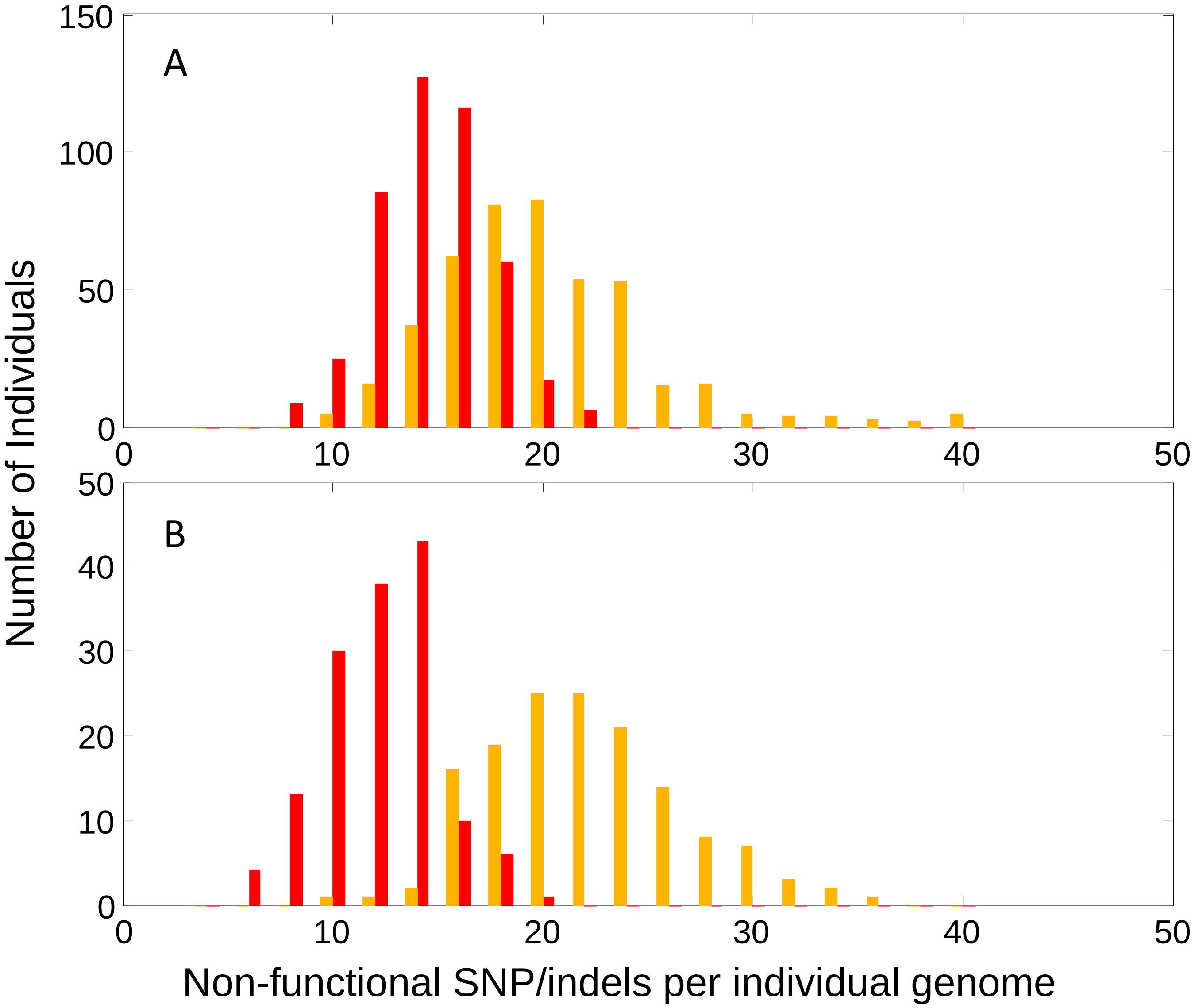

Figure S4: Distribution of deleterious SNP count per individual genome. A) Using the Illumina Golden-Gate technology. B) Using the 1000 Genomes Project data. Red- homozygote loci; orange - heterozygote loci.

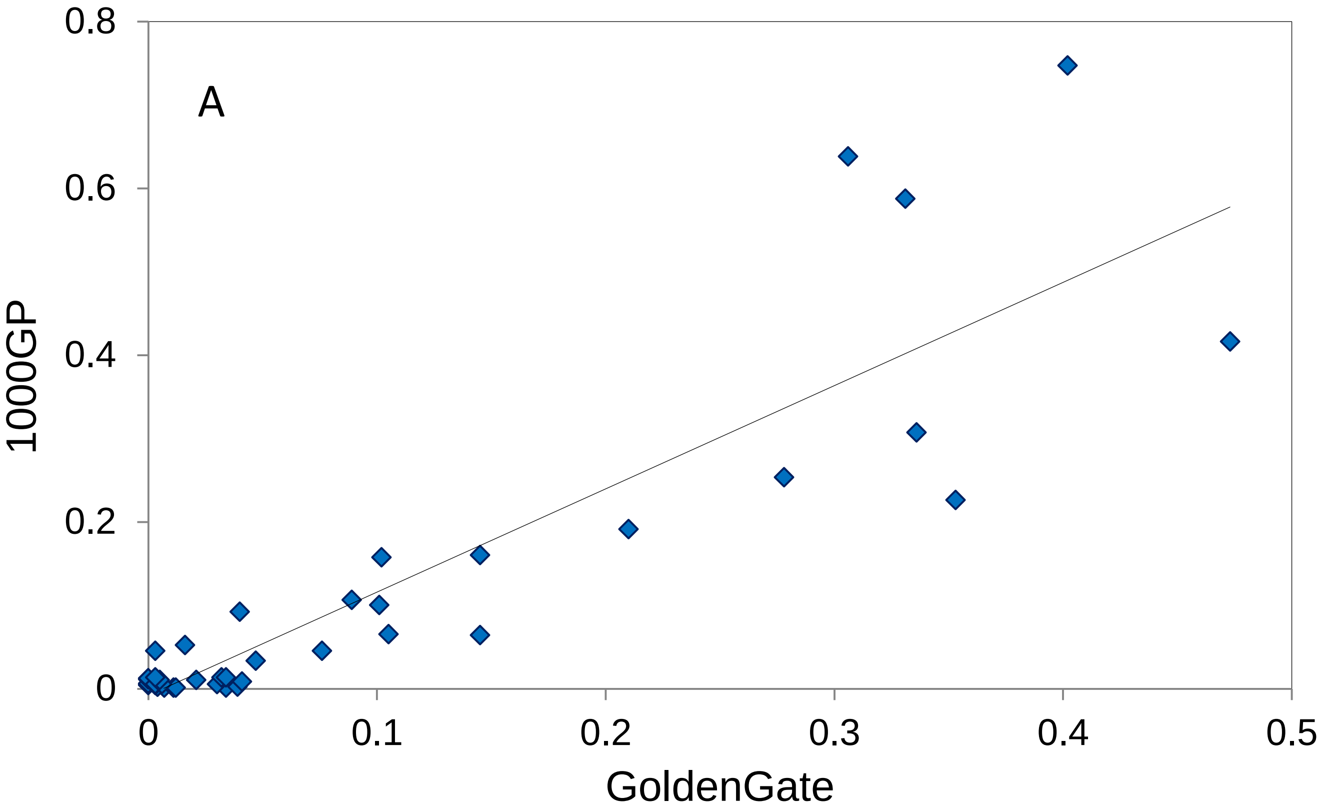

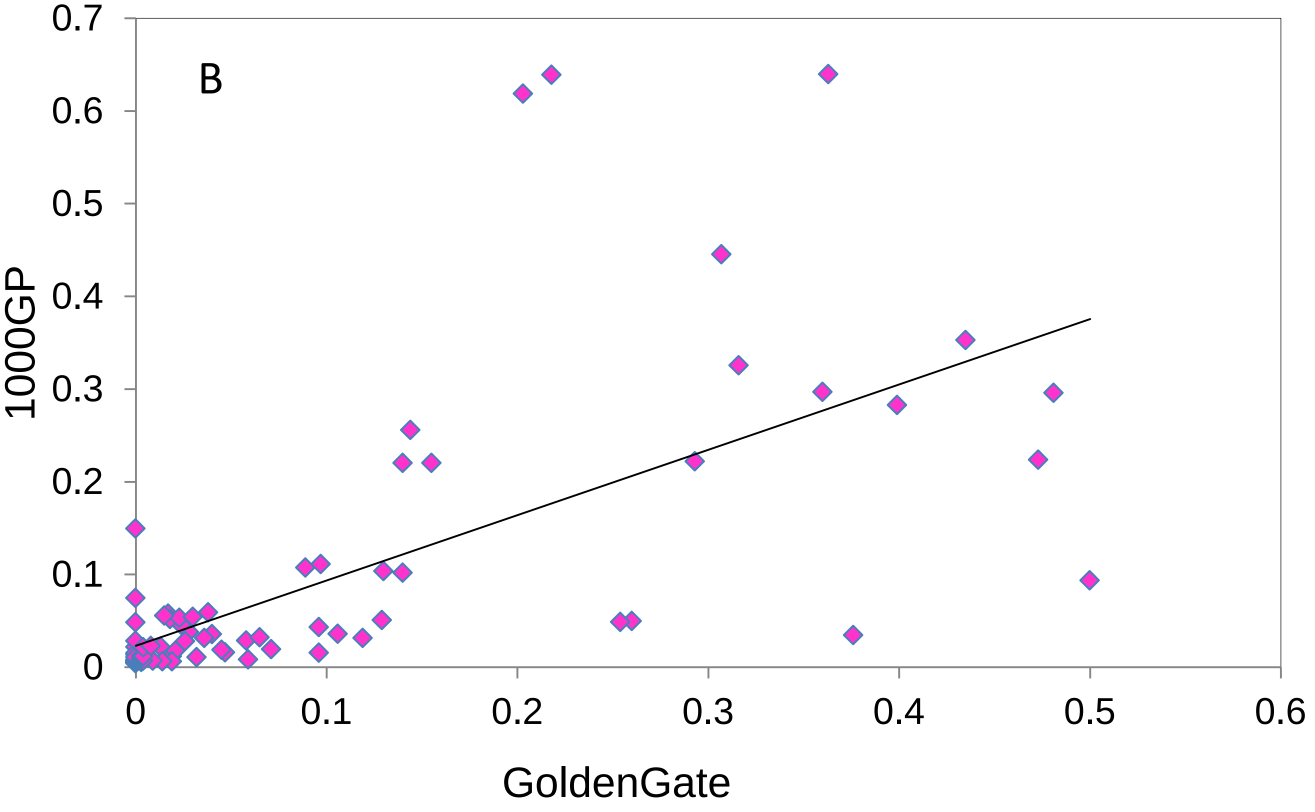

Figure S5: Correlation of the minor allele frequencies measured with the Illumina GoldenGate custom chip (GoldenGate) with the 1000 Genomes Project data (1000GP). A, nonsense SNPs (R^2=0.78). B, indels (R^2=0.44).

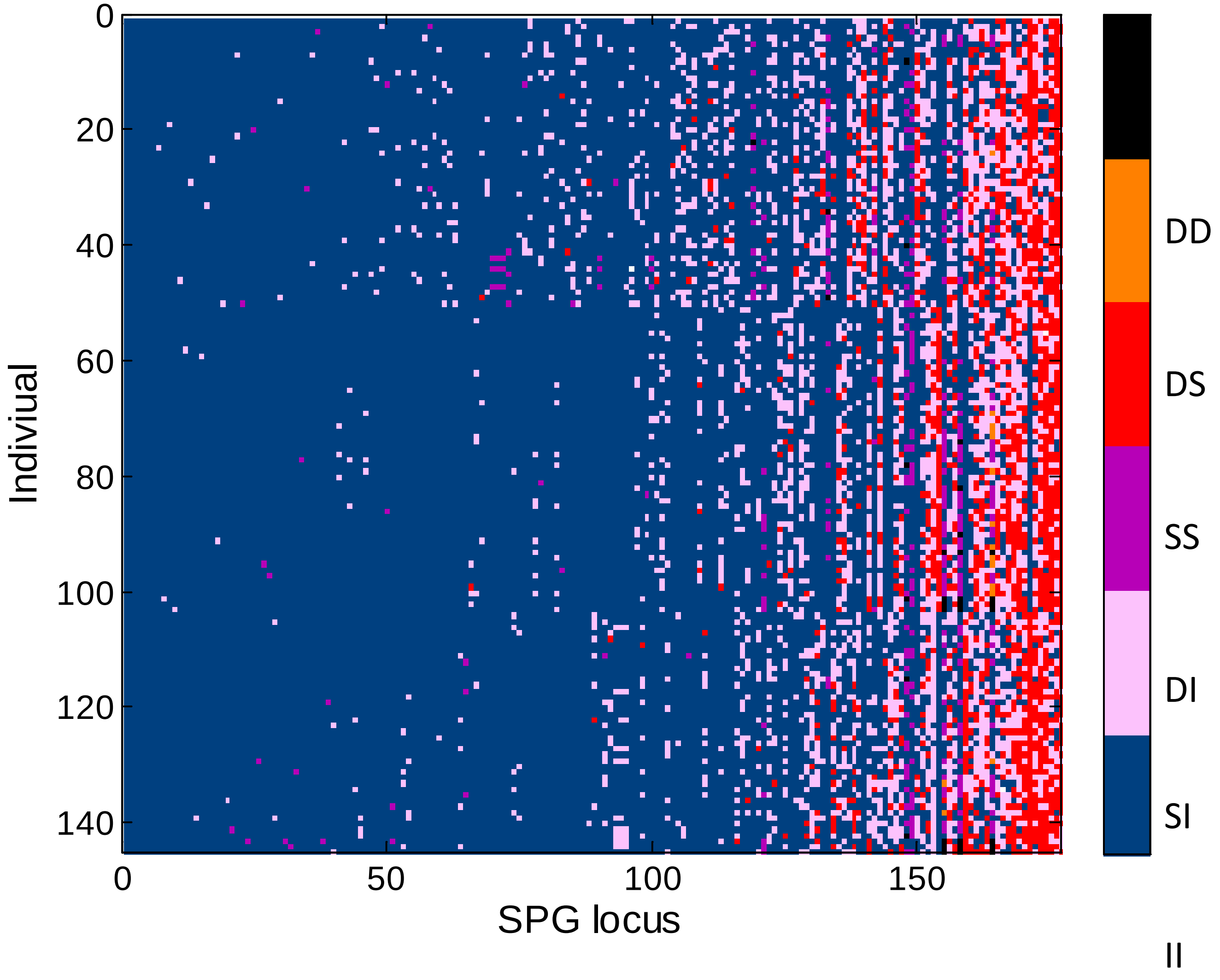

Figure S6: The complete set of nonfunctional allele genotypes in 145 individuals. Individuals 1-50 are Africans, 51-103 are Asians and 104-145 are Europeans. Allele statuses and colors are as in Figure 9.

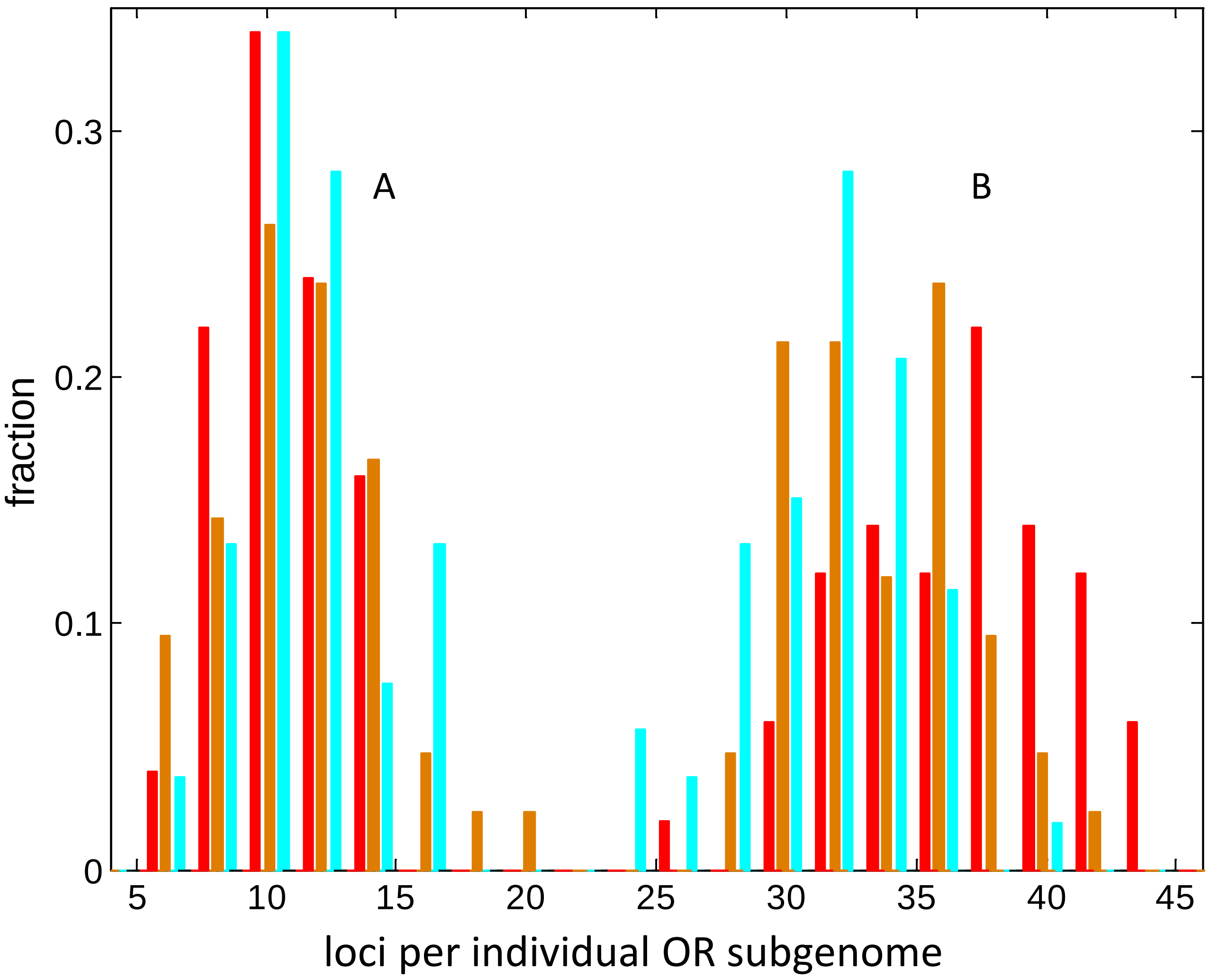

Figure S7: Distribution of disrupted OR count in an individual genome coming from African (red), Asian (light blue) and European (brown) origins. A) homozygously deleted, B) including heterozygote loci.

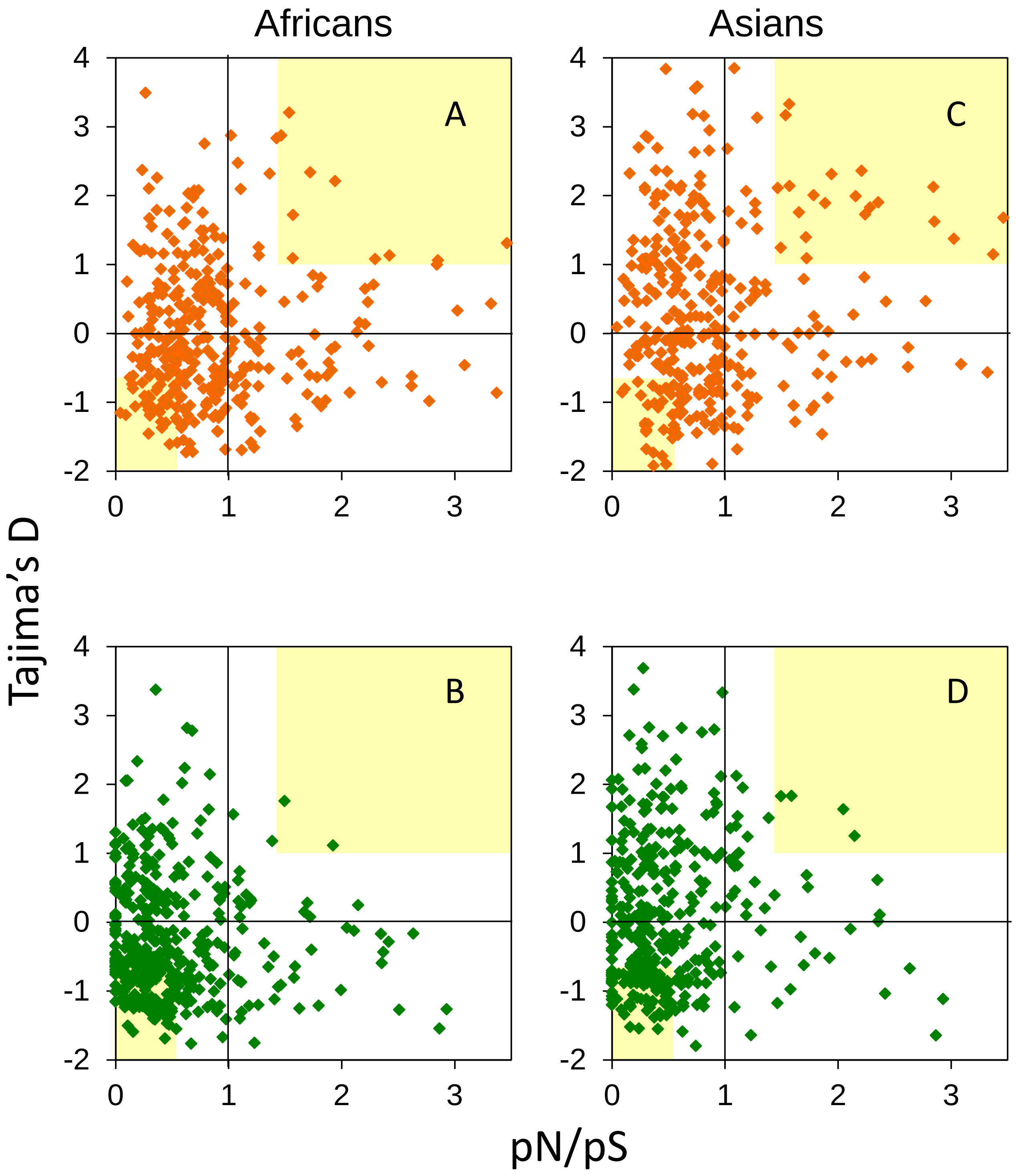

Figure S8: Correlation of non-synonymous to synonymous substitution rate (pN/pS) with Tajima's D values for 364 intact OR genes (A and C) and 439 single coding exon genes (B and D). Data are plotted for the African and Asian populations.

Figure S9: The number of disrupted chromosomes for deleterious OR SNP loci from the 1000 Genomes Project (red), the Illumina GoldenGate experiment (blue) and Exome sequencing data (green). The total intact chromosomes count is shown in grey.
The actual numbers are given in Table S8.

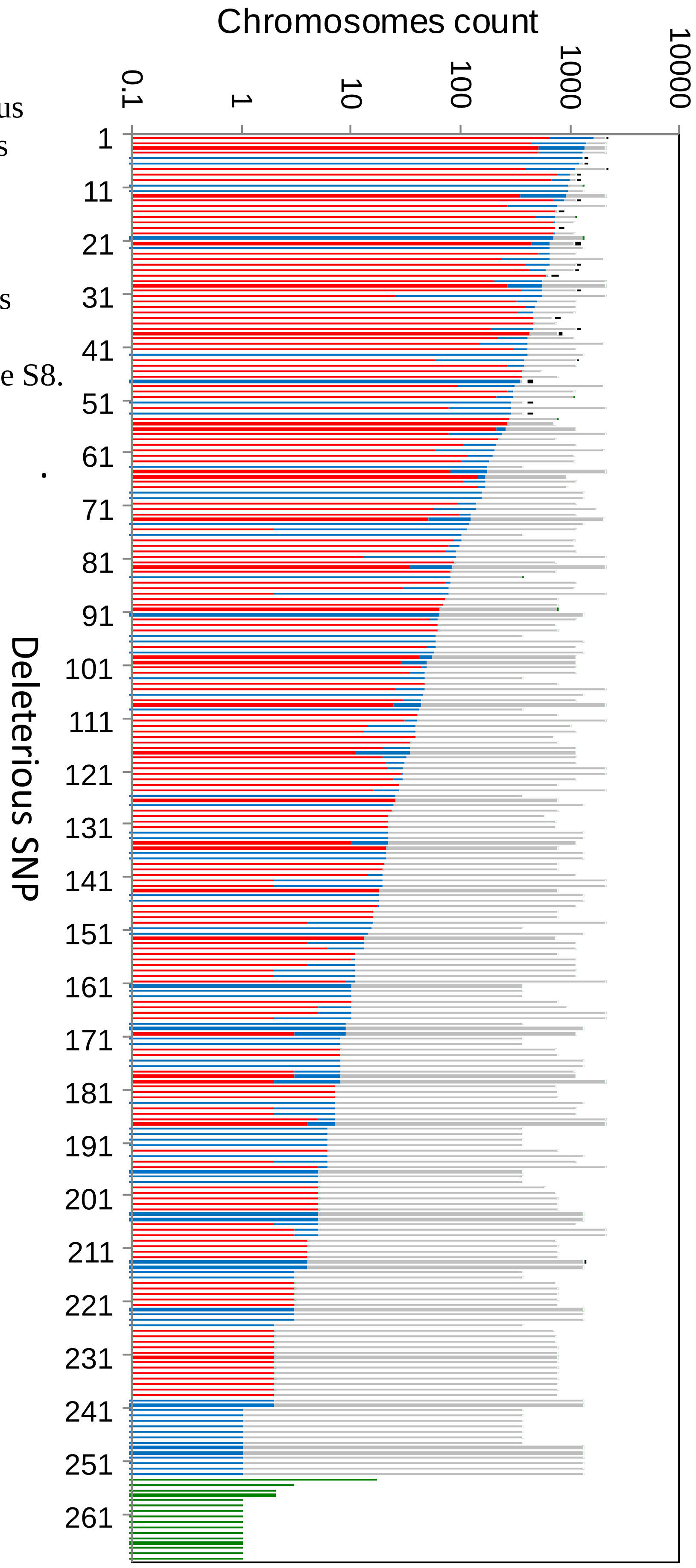

Table S1: Databases and sources used for generating the OR variation compendium. Note that the CopySeq algorithm was reapplied in the current study using ~10kb intervals (coding exon, 7kb from the 5', and 2kb from the 3').

	Source	Type of variation	Version	Genome	URL
**1**	dbSNP [1,2]	SNPs, indels	dbSNP#131	hg19	http://www.ncbi.nlm.nih.gov/snp
**2**	1000 Genomes Project [3]	SNPs	20100804	hg19	http://www.1000genomes.org/
**3**	1000 Genomes Project[3]	Indels	2010_07	hg18	http://www.1000genomes.org/
**4**	Personal genomes: C. Venter [4], J. Watson [5], NA12878 [3],NA12891[3], NA12892 [3],NA19240 [3],NA18507[6]	SNPs		hg19	
**5**	NGS Exome data	Deleterious SNPs		hg18	
**6**	Published study [7]	Deleterious SNPs		hg18	
**7**	NGS targeted sequencing of 20 ORs, validated by Sequenom [8]	Deleterious SNPs		hg18	
**8**	Database of genomic variants (DGV)	Deletions	V9_march2010	hg19	http://projects.tcag.ca/variation
**9**	CopySeq [9]	Deletions	3kb, 10kb	hg18	
**10**	Custom hybridization array data [10]	Deletions		hg18	
**11**	MoDIL [11]	Deleterious SNPs		hg18	http://compbio.cs.toronto.edu/modil
**12**	Integrated SV map of the 1000GP consortium [12]	Deletions		hg18	http://www.1000genomes.org/
**13**	NGS of fosmids [13]	Deletions		hg18	http://hgsv.washington.edu/

References:

1. Day IN (2010) dbSNP in the detail and copy number complexities. Hum Mutat 31: 2-4.
2. Bhagwat M (2010) Searching NCBI's dbSNP database. Curr Protoc Bioinformatics Chapter 1: Unit 1 19.
3. Consortium. GP (2010) A map of human genome variation from population-scale sequencing. Nature 467: 1061-1073.
4. Levy S, Sutton G, Ng PC, Feuk L, Halpern AL, et al. (2007) The diploid genome sequence of an individual human. PLoS Biol 5: e254.
5. Wheeler DA, Srinivasan M, Egholm M, Shen Y, Chen L, et al. (2008) The complete genome of an individual by massively parallel DNA sequencing. Nature 452: 872-876.
6. Bentley DR, Balasubramanian S, Swerdlow HP, Smith GP, Milton J, et al. (2008) Accurate whole human genome sequencing using reversible terminator chemistry. Nature 456: 53-59.
7. Menashe I, Man O, Lancet D, Gilad Y (2003) Different noses for different people. Nat Genet 34: 143-144.
8. Hasin-Brumshtein Y (2010) Genetic variation in human olfactory receptors: from evolution to olfactory sensitivity [PhD]. Rehovot: The Weizmann Institute of Science.
9. Waszak SM, Hasin Y, Zichner T, Olender T, Keydar I, et al. (2010) Systematic inference of copy-number genotypes from personal genome sequencing data reveals extensive olfactory receptor gene content diversity. PLoS Comput Biol 6: e1000988.
10. Hasin-Brumshtein Y, Lancet D, Olender T (2009) Human olfaction: from genomic variation to phenotypic diversity. Trends Genet 25: 178-184.
11. Lee S, Hormozdiari F, Alkan C, Brudno M (2009) MoDIL: detecting small indels from clone-end sequencing with mixtures of distributions. Nat Methods 6: 473-474.
12. Mills RE, Walter K, Stewart C, Handsaker RE, Chen K, et al. (2011) Mapping copy number variation by population-scale genome sequencing. Nature 470: 59-65.
13. Kidd JM, Cooper GM, Donahue WF, Hayden HS, Sampas N, et al. (2008) Mapping and sequencing of structural variation from eight human genomes. Nature 453: 56-64.

Table S2: Twenty-five SPGs selected to represent the highest inter-population variability, with the frequency of inactive allele in each population.

	**Africans**	**Asians**	**Europeans**
OR1E3P	**0.45**	**1.00**	**0.89**
OR2T12	0.00	**0.43**	0.20
OR2T32P	**0.66**	**1.00**	**1.00**
OR2T35	0.00	**0.92**	0.00
OR2T5	**0.12**	**0.29**	0.08
OR4C5	**0.16**	**0.62**	**0.48**
OR4K3P	**1.00**	**0.58**	0.13
OR4N2	0.00	**0.37**	0.00
OR4S2	0.09	**0.36**	**0.25**
OR4X2	**0.41**	**0.16**	**0.13**
OR5K3	0.18	0.00	0.44
OR5K4	**0.24**	0.00	**0.46**
OR5P2	**0.21**	0.08	0.02
OR6C1	0.10	**0.41**	**0.36**
OR6Q1	**0.15**	0.03	**0.21**
OR9A4	**0.22**	0.08	0.10
OR10AC1P	0.00	**0.42**	**0.18**
OR10J4P	**0.39**	0.01	0.04
OR13C2	**0.48**	0.03	**0.17**
OR13C5	**0.70**	0.00	**0.17**
OR51F1	**0.56**	0.00	**0.15**
OR51I1	0.00	**0.31**	0.02
OR51I2	**0.17**	0.00	**0.37**
OR51J1	**0.41**	0.00	**0.11**
OR52E1P	**0.38**	**1.00**	**0.49**

Table S3: OR genes suspected to be under purifying selection.

OR gene	Tajima's D in Africans	Tajima's D in Asians	Tajima's D in Europeans	dN/dS
OR10AG1	-1.61	-1.90	-0.13	0.48
OR5AS1	-1.46	-1.33	0.11	0.29
OR4F15	-1.37	NA	-1.16	0.41
OR2L5	-1.30	-1.78	-1.44	0.45
OR6T1	-1.29	1.00	0.45	0.40
OR13C4	-1.19	-1.68	-1.40	0.30
OR51T1	-1.18	-0.86	-0.60	0.09
OR2K2	-1.17	1.06	0.17	0.31
OR1E1	-1.15	0.09	-1.25	0.04
OR2AK2	-1.14	1.26	0.38	0.39
OR1J2	-1.13	0.58	-0.83	0.39
OR8B8	-1.11	2.08	0.32	0.29
OR10G8	-1.08	-0.28	-1.48	0.33
OR4M2	-1.07	1.37	-0.40	0.40
OR2M3	-1.06	0.98	1.12	0.17
OR5P3	-1.06	1.08	0.71	0.31
OR4K13	-1.03	-0.52	0.23	0.46
OR2Z1	-1.02	NA	0.32	0.26
OR5M3	-1.01	-0.80	0.01	0.47
OR2B6	-0.97	-0.83	-0.99	0.43
OR5M10	-0.94	0.94	1.03	0.30
OR5K2	-0.91	-1.30	-1.05	0.29
OR1N1	-0.91	-0.15	-0.29	0.26
OR4C13	-0.89	0.21	-0.92	0.48
OR13C3	-0.83	-0.76	-0.55	0.36
OR7D2	-0.80	-0.46	NA	0.16
OR5J2	-0.78	NA	-0.40	0.38
OR4Q3	-0.74	-0.44	1.33	0.40
OR12D3	-0.74	0.69	0.22	0.15
OR6C76	-0.71	NA	-0.66	0.24
OR1M1	-0.66	-1.08	-0.24	0.41
OR9A4	-0.64	NA	NA	0.13
OR10P1	-0.62	1.09	1.75	0.30
OR1F1	-0.62	1.75	0.41	0.47

OR2T7	-0.61	-0.31	-1.00	0.16
OR5L2	-0.59	-1.02	-0.56	0.38
OR1G1	-0.51	-1.40	NA	0.30
OR8B12	-0.48	-0.70	-0.27	0.23
OR2L2	-0.38	-0.33	-0.87	0.23
OR4D10	-0.37	-0.25	-1.09	0.21
OR2T27	-0.34	0.17	-0.66	0.15
OR2AJ1	-0.28	-0.79	-1.23	0.48
OR4X2	-0.26	-0.09	-0.76	0.38
OR5V1	-0.23	-0.44	-0.93	0.42
OR4K14	-0.09	-0.99	-0.21	0.45
OR2T6	-0.06	0.21	-0.96	0.50
OR1C1	0.05	-0.15	-1.01	0.31
OR6C65	0.25	-0.80	0.00	0.11
OR10H1	0.38	-1.04	0.81	0.32
OR14A16	0.48	-1.43	-0.16	0.30
OR1F12	0.52	0.47	-0.53	0.31
OR6V1	1.17	-1.31	-0.78	0.32
OR1A1	1.22	-0.90	0.84	0.26
OR2V1	1.44	-1.40	-0.96	0.46
OR11L1	1.79	-1.73	-0.30	0.37
OR51F1	2.26	-1.92	1.97	0.37
OR51E2	NA	0.47	-0.82	0.11
